# Modern medicine and the one‐size‐fits‐all approach: A clinician's comment to Alexandra Pârvan's “Mind Electric” article

**DOI:** 10.1111/jep.13003

**Published:** 2018-08-15

**Authors:** Heinz Katschnig

**Affiliations:** ^1^ Medical University of Vienna Vienna Austria; ^2^ IMEHPS Research Institute for Social Psychiatry Vienna Austria

**Keywords:** diagnosis, health care, patient‐centred care, philosophy of medicine, psychopathology, specialization

## Abstract

As a clinician, I can easily agree with the author that a person's own reality of being healthy is independent of physical evidence or clinical categories and that this perspective should be considered to improve clinical care. However, I cannot follow the assumptions about the nature and working of modern medicine and psychiatry as typically using “black box” and one‐size‐fits‐all treatments in daily practice. I outline several working contexts of doctors where this criticism does only marginally apply or not at all and wonder whether the author might wish, if possible at all from a philosophical viewpoint, to differentiate her concepts with regard to these different contexts. In addition, I think that ill health in the field of psychiatry might have to be dealt with differently than physical ill health.

## 1 INTRODUCTION

Alexandra Pârvan's paper “The mind electric: challenges to clinical categories from a person‐centred perspective and the possibilities of metaphysics and art for clinician, patient, and treatment”^1^ is a rich and inspiring text, touching upon a wide array of topics from philosophy, literature, and history supporting the author's stance that “a person's own reality of being healthy is independent of physical evidence or clinical categories”. While I can agree with this statement, I have to leave specific philosophical and terminological comments to the concept of the “body/mind/person electric” to respective experts. The author's suggestion that “frameworks typical to metaphysics and art could be used in clinical treatment in somatic and psychiatric contexts, to ensure improved care” generates interest in a clinician, given the perennial need to enhance person‐centred care.

As a medical doctor and psychiatrist with life‐long clinical experience “out there”, my comments will focus, perhaps unjustly, on the argument that the image of science‐based medicine and psychiatry depicted and taken as the point of reference of the philosophical deliberations of the paper refers to only a part of the spectrum of clinical practices. To start with, I fully agree that “working only with rigid, standardized, scientific, normatively defined frameworks for clinical care is not the best way to care for patients”. But I doubt that “treatment approaches focused on clinical categories, disease, provision and promotion of standardised or ‘black‐box’ therapies” are actually that dominant in daily practice and I do not think that “overreliance on science” is the rule out there. When reading the article, I was inadvertently reminded of George Bernhard Shaw's play “The Doctor's Dilemma”, where he satirically depicts doctors competing for the correct diagnosis of their “case”, with one character exclaiming: “The case is as plain as a pikestaff: bad blood‐poisoning.”^2^

My main conclusion is that the complexities of today's medicine and the broad spectrum of working contexts, in which patients and doctors (and also other staff) meet, might have to be considered in more detail to increase the chances that the ideas presented in the paper find acceptance in the medical community and contribute to “improved care”. I shall provide some thoughts on this issue in the first part of my comments, which focuses on medicine in general. In the second part, I will more specifically deal with psychiatric issues.

## 2 MEDICINE IS A COMPLEX UNDERTAKING WITH MANY DIFFERENT CONTEXTS OF THE DOCTOR‐PATIENT RELATIONSHIP

The starting point of Alexandra Pârvan's philosophical considerations is a fundamental critique of clinical or scientific medicine, as it is taught in universities (medical schools), variably also called orthodox, conventional, or school medicine. This type of medicine, as the author says, regards the sick person as affected by a category of something irregular and negative, the disease, which inhabits a person and has to be removed using the instruments of “diagnosis” and corresponding “treatments” in a one‐size‐fits‐all approach. According to the author, this approach to a sick person not only neglects the way people experience “ill health” (as I cautiously say here, since terms used in this context are a minefield of misunderstandings) as integrated in their person, but also prevents a person suffering from such ill health to experience their ill health in an integrated way.

Critique of modern medicine is not new ‐ many kinds of “alternative medicine” have been promoted and described as person‐centred, holistic, and more “humane” than clinical medicine, ranging from traditional folk medicine and shamanism to homoeopathy and other alternative approaches. They regard themselves, as the overall term says, as “alternatives” to scientific medicine. The interesting idea presented by the author of the “Mind Electric” article is not to develop an alternative system of medicine but to integrate the concept of the “body/mind/person electric” into clinical practice to “ensure improved care”.

My response as a clinician is as follows: While in medical schools the criticized approach is certainly taught, reflected in medical textbooks, conference presentations, and clinical guidelines published by professional medical associations, furthermore, while medical research and the development of new treatments (especially pharmacological ones) work with categorical diagnoses, and while many payment systems for doctors require to name specific diagnoses and interventions, I suggest, based on my own experiences and that of many of my colleagues, that “clinical reality”, ie, the situations where doctors and patients meet in daily practice, is in many instances different from what the author criticizes. My hypothesis is that the “scientific medicine” described by the author is rather something presented by professional medical organizations to the outside world for showing how “professional” they are. I present below four (slightly overlapping) observations concerning the variety of situations in clinical practice.

### 2.1 Clinical categories are more important during medical education than in actual daily practice

After leaving medical school and being confronted out there on my own with persons coming to me with different states of physical and psychological suffering, I quickly realized that what I had been taught was not of that much use as expected. Most of my patients did not clearly correspond to a specific diagnosis, and I had not been taught the skills of dealing with uncertainty. I had to start afresh (with the acquired knowledge in mind though) and thought of the old managerial wisdom “Who can, does, who can't, teaches.” In other words, you start afresh after university according to the principle of “learning by doing”. The clinical categories became less important with the fuzzy and unclear situations in daily practice—although, to avoid malpractice allegations, they were always there in the background, but not as dominant as one might think. Adherence to clinical guidelines in daily practice is poor,^3^ and doctors faced with nonuniform clinical problems decry guidelines with rigid rules about what is appropriate, as “cookbook medicine”.^4^ The articles referenced here are from 1999 and have been cited in the literature until today several thousand times. The issue is still hot.

### 2.2 Clinical categories are more relevant in acute and emergency situations than in long‐term care provided by general practitioners

Depending on the period of time a doctor is involved with his patients, there exists a large array of working situations, ranging from those particular to doctors working in acute and emergency services, over those specific to doctors involved in specialized medical assessments and interventions for short time periods, to those characteristic of doctors accompanying patients with chronic diseases. The first two probably correspond more to the one‐size‐fits‐all approach than the latter, typically a primary care doctor, who can be a companion to his patients and may tend to rather deal with the patient as a person; he knows his patients' illness behaviour and lifestyle, has to adapt constantly his therapeutic strategies (not only the type and dosage of medication) and must be flexible. John Berger, an English novelist, painter, and poet, has portrayed in a wonderful essay^5^ the life of a Scottish country doctor, showing how physical and psychological intimacy is central to his relationship with his patients and ‐ given the ambiguities of scientific medicine and the naming of illnesses ‐ what the meaning of “good” doctoring in the context of general practice could be. Many students might have had similar ideas about their future professional profile at the time when they chose to study medicine. The book was written 50 years ago, and it is true, this species of doctors is in danger of getting extinct today, with young doctors striving more often to become specialists than generalists.

### 2.3 Specialization enhances the use of clinical categories and one‐size‐fits‐all approaches

An important role in today's clinical medicine is played by specialists. Medical specialty disciplines have developed over the last 150 years or so, hand in hand with the scientific progress of medicine, with medical knowledge becoming increasingly large and varied so that no single doctor can have all necessary information and skills anymore. Today's medical specialists know more and more of an always smaller aspect of medicine. Radiologists and some types of surgeons are extreme examples for such doctors having the tendency to see their job as diagnosing a disease that has entered the patient, or as fixing, removing, or replacing diseased parts of the body, and might have no time to care about the person. I vividly remember when I was referred by my GP to a radiologist for a lung X‐ray, arriving late because of a traffic jam and being the last patient for whom they had waited. While she thought I was at the toilet, the assistant shouted to the radiologist, who was in another room, “the lung is here.” I was rather amused than offended, because this is obviously the jargon they use to organize their “patient processing”. I must say, the more lungs a radiologist sees, or the more thyroid operations a surgeon performs, the more I trust their skills. The modern division of labour has led to this type of specialist, who sees the patient for a short intervention only in his specific field of competence, which is especially the case in hospitals (where, by the way, most of the time other staff than doctors are the patients' contact). Within medicine, there exists criticism of this “fragmented” care and attempts for improving “continuity of care” and “coordinated care” have sprung up, but the obstacles are perceived more on the organizational, legal, and financing levels (eg, paying for time rather than for interventions) than in the issue of disease categories. The development of specialized medicine is irreversible—there will never again exist a universal genius like Hippocrates or Paracelsus.

### 2.4 It matters what kinds of ill health the doctor has to deal with

There are different kinds of ill health. There are patients with clear‐cut diseases who justifiably receive a clear diagnosis from school medicine and a corresponding treatment ‐ some specific infectious diseases correspond to this model, but also many other diseases. Following the movement of evidence‐based medicine to provide systematic reviews, clinical guidelines have been produced by professional medical associations for these clear‐cut categorical diseases, but as already mentioned, they are often not followed in practice.^3^ A large proportion of patients, especially the ever‐increasing number of elderly patients, suffer from multiple diseases, and guidelines how to deal with such combinations (called “multi‐morbidity” by scientific medicine) are not that clear‐cut or do not exist at all, and within the limits of the professional principle of “nil nocere”, many doctors will use a kind of trial and error approach in such situations. Finally, especially in primary care, a very large proportion of patients suffer from symptoms that cannot be classified at all as a specific disease, and in this fuzzy situation, characterized by uncertainty, the doctor cannot use a one‐size‐fits‐all approach.

In sum, I would rather provocatively say that clinical medicine is not a science as such, but a profession that is only partly based on science. Medicine as a profession can be looked at with the eyes of the sociology of professions. From this viewpoint, professions in general are characterized by ownership of a specialized body of knowledge and skills, which defines the field of competence and the scope of potential clients, including the demarcation from other professions; holding a high status in society (both through financial and other rewards); being granted autonomy (and thereby power) by society, eg, in recruiting and excluding members; and being obliged, in return for the above, to guarantee high‐quality standards in providing services (being “professional”) and following ethical rules.^6^ “Ownership of a specialized body of knowledge and skills” is the central point here, both inwardly for increasing knowledge and socializing its trainees and outwardly for justifying the existence of the profession. But actual daily practice of “professionals” can be much different from what the profession declares as standard knowledge.

## 3 PSYCHOPATHOLOGICAL PHENOMENA THAT ARE FUNDAMENTALLY DIFFERENT FROM PHYSICAL PATHOLOGY

In addition to the comments above regarding medicine in general, I present below a few thoughts concerning psychiatry, my personal professional field, and its patients. Again, I think that differentiation might be appropriate in several respects—basically my suggestion is that “the person's own way of being healthy” is specifically “coloured” by different psychopathologies and not analogue to situations with just physical ill health, and that most working contexts of psychiatrists make it difficult to use a one‐size‐fits‐all approach. I divide my arguments again into four (slightly overlapping) observations.

### 3.1 Psychopathological states are experienced differently than somatic illnesses are

Experiencing “one's own reality of being healthy as independent from clinical categories” has certainly to do with what could be cautiously called a “psychological” component, which where physical health is considered, is admittedly different for each person but not regarded as deviating from the normal (however that is defined). It is my conjecture that “psychopathological states” interfere with how one's own health is experienced. In other words, it is difficult for me to imagine here a complete analogy between physical and mental ill health, ie, to agree with the author's assumption that “health is not different for psychiatry patients than it is for somatic patients.” Also, most “psychiatric patients” do not see their problems primarily as health problems, at least before they enter the realm of clinical psychiatry. While I agree with the author that categorical psychiatric diagnoses are problematic,^7^ this is not relevant here. I give a few examples, showing also how diverse such “abnormal” psychological problems are: Persons with manic behaviour usually see their state not at all as an illness; patients with obsessive compulsive symptoms experience a lack of personal freedom since they find themselves forced to think or act repeatedly in the same way, which destroys all their personal life; persons with delusions of persecution feel tormented by their pursuer and do not regard their wrong perception as a disease but as reality; and depressed persons may feel unduly guilty of neglecting their family or their job and, seeing their situation as a personal failure, may commit suicide. Even without entering psychiatry and receiving perhaps a clinical diagnosis, such psychopathological states *are* experienced by people. From a clinical viewpoint, it might be an interesting task to elaborate on the “person's own realty of being healthy” in relation to these and other different psychopathological experiences.

### 3.2 Joint presence of physical and mental pathology

I would like to raise an additional point (closely related to my thoughts above, but still different): It would be interesting to think of the not so rare situation that a patient experiences both mental and physical ill health concomitantly (clinicians call it “comorbidity”). Many studies show that persons with diabetes often suffer from depression and that patients with severe mental illness have a higher risk for developing physical illnesses.^8^ It would be interesting to consider the implication of the presence of ill health in both domains for the “person's own realty of being healthy”.

### 3.3 Psychiatry is in itself multifaceted, which has to be considered when criticizing diagnostic and clinical categories and the concept of a brain disease

Another issue to consider is that psychiatry in itself is not uniform. And it is not just subspecialties (as in surgery), but it is different philosophies. “Biological psychiatry”, at which the major part of the article seems to target, is a specific field of psychiatry, more frequently found in universities, where drug companies fund clinical trials, and the network of such psychiatrists promulgates the use of drug treatment of mental disorders. Correspondingly, they favour a brain disease model of mental disorders. But this is not of much help in daily practice, where patients already come with their own views to the psychiatrist, some cherishing medication, but many rejecting it out of several reasons,^9^ an important one being that they do not see their problem as a “health problem”. Psychiatrists take (or better said “have to take”) these patient expectations into account. Also, in clinical practice, medication is often used in the sense of helping to decrease vulnerability to life stress and not as treating a “disease” in a “black box” way. Many psychiatrists, in addition to their medical knowledge, may have also acquired psychotherapeutic skills (often after their university training), may also consider life events,^10^ strengths and disabilities of their patients in everyday life (in a multiaxial assessment approach in addition to clinical diagnosis^11^), and take care of quality of life issues,^12^ when assessing the problems of their patients and attempting to assist the patients in solving them. Finally, when thinking of the many practice fields of psychiatry, it has to be considered that the mental health care systems in many Western countries offer so‐called community mental health services with multiprofessional teams in ambulatory and mobile care settings, in which it is much more difficult to neglect the patient's personal reality than, say, in a psychiatric hospital or in a psychiatric office. These different contexts of psychiatric working situations, similarly to those mentioned above for general medicine, might have to be taken into consideration in philosophical deliberations intending to improve clinical care.

### 3.4 The utility of categorical psychiatric diagnoses is being increasingly questioned by professional psychiatry itself

As one example for this trend, I am quoting here the editorial of the latest edition (May 2018) of the worldwide leading psychiatric journal *World Psychiatry* of the World Psychiatric Association, entitled “Why the clinical utility of diagnostic categories in psychiatry is intrinsically limited and how we can use new approaches to complement them”.^13^ Alexandra Pârvan is right when she critically enumerates the many different diagnoses given to van Gogh by different psychiatrists and neurologists, but it has to be noted that except for his personal doctors in Arles and Saint Remy (at a time when modern classification systems did not exist), these psychiatrists had never seen or interviewed van Gogh, but derived their diagnoses much later in a kind of “detective” approach from reports about van Gogh's behaviour—and these reports are, of course, limited in scope and depth. In modern terms, one would say that the reliability of the diagnoses given to van Gogh is poor. Modern psychiatry has managed by using so‐called operational diagnostic criteria to increase this reliability, but validity is still poor, ie, the issue whether the definition describes a real disease, a “natural kind”.^7^ The surprisingly new development is that now even the “utility” of categorical diagnoses is questioned by the profession itself.

## 4 CONCLUSION

I am aware that, because of my lack of philosophical expertise, I have not done justice to the breadth and depth of Alexandra Pârvan's article by focusing in my comments on clinical issues. From a clinical viewpoint, there are certainly medical doctors and psychiatrists who behave as the author says—seeing the ill health of the patient as a disease entering the patient that needs to be removed and fixed with a one‐size‐fits‐all approach. But there are also others, and the suggestion is that the approach used by a doctor may depend a lot on the actual working situation or the role a doctor finds himself in in the health care system. Also, the different kinds of ill health a doctor is confronted with matter for which approach he is using—ranging from the clear‐cut to the fuzzy, from the physical to the mental with many different psychopathologies. It would be interesting to analyse the concept of the “body/mind/person electric” in relation to the different contexts and concepts discussed above, considering different roles of doctors, working situations, and pathological phenomena, and perhaps also including other types of professionals in the health care field, with whom doctors often work in a team. I guess this differentiation is needed if the ultimate goal is to win over clinicians and demonstrate that philosophical reasoning can help them to improve clinical care in a person‐centred paradigm.
